# Association between Lifetime Classic Psychedelic Use and Sick Leave in a Population-Based Sample

**DOI:** 10.3390/ijerph191811353

**Published:** 2022-09-09

**Authors:** Christin Mellner, Micael Dahlen, Otto Simonsson

**Affiliations:** 1 Department of Psychology, Stockholm University, 114 19 Stockholm, Sweden; 2 Stockholm School of Economics, 114 19 Stockholm, Sweden; 3 Department of Clinical Neuroscience, Karolinska Institutet, 171 77 Stockholm, Sweden; 4 Department of Sociology, University of Oxford, Oxford OX1 1JD, UK

**Keywords:** health economics, LSD, psilocybin, psychedelics, public health, sickness absence, sickness absenteeism

## Abstract

Objectives: Absenteeism from work due to illness, and related costs, has increased steadily during the past decades. In recent years, there has been a reemergence of research on the therapeutic effects of classic psychedelics showing associations with both physical and mental health. However, the association between classic psychedelics and sick leave remains unknown. The aim of this study is to investigate the association between lifetime classic psychedelic use and sick leave in the past 30 days among adults in the United States (*N* = 407,717), using data from the National Survey on Drug Use and Health (2005–2019), weighted to be representative of the US adult population. Methods: The primary analysis was conducted using multiple linear regression, controlling for sociodemographic characteristics, risky behavior, and use of other substances. Results: There was a significant and negative association between lifetime classic psychedelic use and sick leave in the past 30 days (B = −0.09, *p* < 0.01) when adjusting for all control variables. Conclusion: These findings suggest that classic psychedelics could potentially lead to reduced sick leave and associated costs in the general population, but more research is needed to investigate potential causal pathways of classic psychedelics on sick leave and evaluate possible mechanisms.

## 1. Introduction

Improving health globally is a vital social objective as this is associated with better and longer lives [1]. Health also has an influence on individual income growth through increased labor market participation and productivity [2,3] and on investment in education and innovation [4]. Meanwhile, absenteeism from work due to illness is steadily increasing, as are related sickness and health care benefits, lost productivity, labor turnover costs, and the disability burden.

There are both direct and indirect costs of ill-health and disease for the economy and society and sick leave serves as a means to measure these costs [5,6,7,8]. In the United States (US), employers’ annual cost for employee sick leave in 2015 was $225.8 billion [9], and in 2019 it had increased to $575 billion [10].

The reason for employee sick leave is, generally speaking, the incapacity to work due to illness [11], and sick leave is often regarded as equivalent to ill-health and disease both in the public debate and within research. However, illness does not necessarily carry with it reduced work capacity, and conversely, reduced work capacity can also occur without the presence of illness. Nonetheless, sick leave has been put forth as the immanent result of ill-health and disease [12], which is supported by the fact that the most frequent reasons for sick leave are mental health problems and chronic diseases [13,14,15,16].

### Classic Psychedelics and Health

In the past two decades, research into the therapeutic potential of classic psychedelics for health have reemerged. Classic psychedelics are partial or full agonists at the serotonin 2A receptors and typically include N,N-dimethyltryptamine (DMT), the DMT-containing admixture ayahuasca, psilocybin, lysergic acid diethylamide (LSD), mescaline, and the mescaline-containing cacti peyote and San Pedro [17,18]. In contrast to use of other substances, such as alcohol, cocaine, and heroin, classic psychedelics are considered to carry relatively low medical and psychological risk [19]. In addition, research suggests that classic psychedelics have low risk of dependence compared to other substances such as alcohol, opioids, benzodiazepines or stimulants such as amphetamine and cocaine [20]. In fact, studies have shown that classic psychedelics may have anti-addictive properties [21,22,23,24]. Moreover, previous research suggests that classic psychedelics have immunomodulatory and anti-inflammatory properties [25,26,27], low physiological toxicity [22,28], and appears safe in clinical trials, at least when potential risk populations are screened out [17,29]. Recent clinical trials also suggest that classic psychedelics may be effective in the treatment of various psychiatric disorders, when administered by health professionals in a safe and supportive setting [30,31,32,33,34,35,36].

Findings from clinical research on classic psychedelics are supported by results from cross-sectional studies with nationally representative samples [37,38,39,40], but more recent cross-sectional research has also found associations between lifetime classic psychedelic use and lower odds of obesity and cardiometabolic diseases in the past year [41,42,43]. While causality has not been established, such findings could potentially be explained by healthy lifestyle changes after classic psychedelic use [44,45,46,47].

In light of the growing evidence on the potential link between classic psychedelic use and mental and physical health, as well as the limited knowledge in general on the potential influence of overall substance use on sick leave [48], it is important to better understand possible associations with sick leave, and subsequent work-related costs. Using a representative sample of the US adult population, the present study therefore examined the association between lifetime use of classic psychedelics and sick leave in the past 30 days. We hypothesized that lifetime classic psychedelic use would be associated with lower sick leave in a covariate-adjusted model.

## 2. Methods

### 2.1. Data and Study Population

The present study used pooled data from the NSDUH survey years 2005 to 2019, which contained responses from 407,717 (unweighted) adults aged ≥18 years. The NSDUH is a nationally representative survey of the civilian noninstitutionalized population in the United States conducted each year in all 50 states and the District of Columbia. The NSDUH does not include institutionalized populations such as persons in jails, mental institutions, nursing homes, and long-term care hospitals, or people experiencing homelessness who do not use shelters, and active military personnel. The NSDUH public use data files are available on their homepage.

### 2.2. Variables

The dependent variable was number of days missed work due to illness or injury during the past 30 days (variable names: WORKDAYS (survey years 2005–2014) and WRKSICKMO (survey years 2015–2019)). Consistent with previous research [37], the independent variable was lifetime classic psychedelic use: having ever, even once, used DMT, ayahuasca, psilocybin, LSD, mescaline, peyote or San Pedro (1 = yes, 0 = no).

Both lifetime classic psychedelic use and sick leave have been associated with certain sociodemographic characteristics and use of other substances [11,38,44,48,49]. We therefore controlled for: age, gender, ethnoracial identity, educational level, annual household income, marital status, risky behavior (i.e., “Like to test yourself by doing risky things”), lifetime use of cocaine, other stimulants, sedatives, tranquilizers, heroin, pain relievers, marijuana, phencyclidine (PCP), 3,4-methylenedioxymethamphetamine (MDMA/ecstasy), inhalants, smokeless tobacco, pipe, cigar, and daily cigarettes, as well as age of first alcohol use.

### 2.3. Statistical Analyses

Descriptive statistics (weighted percentages) were calculated for the number of days missed work due to illness or injury during the past 30 days. Two linear regression models were performed to investigate the association between lifetime classic psychedelic use and sick leave in the past 30 days. Model 1 did not control for any potential sources of confounding; Model 2 controlled for sociodemographic characteristics, risky behavior, and lifetime use of other substances. We conducted sensitivity analyses for Model 2 with respondents who were employed full time or part time, respectively. Additional sensitivity analyses were also conducted for Model 2 with lifetime classic psychedelic use divided into the main classes of classic psychedelics: tryptamines (DMT, ayahuasca, or psilocybin), LSD, and phenethylamines (mescaline, peyote, or San Pedro). The analyses were conducted using Stata, version 16.

## 3. Results

The weighted percentage of 0 days of sick leave in the past 30 days was 79% among lifetime classic psychedelic users and 76% among those who did not report lifetime classic psychedelic use (see Simonsson et al., 2021 [42] for weighted descriptive statistics of characteristics of lifetime classic psychedelic users versus non-lifetime classic psychedelic users).

Two linear regression models were performed (see Table 1). The results from Model 1, where no control for potential confounders was made, showed a positive association between lifetime classic psychedelic use and sick leave in the past 30 days (B = 0.11; *p* < 0.01). The results from Model 2, adjusting for all the control variables, showed a negative association between lifetime classic psychedelic use and sick leave in the past 30 days (B = −0.09; *p* < 0.01). Hence, the hypothesis that lifetime use of classic psychedelics would be associated with lower sick leave in the covariate-adjusted model was supported. Sensitivity analyses for Model 2 showed negative associations between lifetime classic psychedelic use and sick leave in the past 30 days both when analysing respondents who were employed full time (B = −0.07; *p* < 0.01) and part time (B = −0.18; *p* < 0.05), respectively. Additional sensitivity analyses for Model 2 showed a negative association between both lifetime tryptamine use (B = −0.09; *p* < 0.01) and lifetime LSD use (B = −0.08; *p* < 0.05), respectively, and sick leave in the past 30 days. No association was observed between lifetime phenethylamine use and sick leave in the past 30 days.

As a thought experiment, we used the results from Model 2 to estimate the unique association between lifetime classic psychedelic use and lower sick leave costs in the US, per the cost reported by Integrated Benefits Institute (2020): $575 billion. A 30-day period could comprise 20 to 22 work days, depending on the calendar. Under the assumption that the effect is uniformly distributed over a) the working population and b) all workdays of the year (12 30-day periods), one could estimate the unique association between lifetime classic psychedelic use and lower sick leave costs in the US as:

B × 12 × TC/max WD: min WD(1)

where B (−0.09) is the relative association of sick leave in the past 30 days among lifetime classic psychedelic users, *TC* ($575 billion) is the total annual cost of sick leave in the US, *max WD* (264) is the absolute maximum derived number of work days in a year, 22 per 30-day period × 12, *min WD* (230) is the absolute minimum derived number of work days in a year (20 per 30-day period × 12 – 10 paid holidays).

From this equation, based on the findings from Model 2, the estimated unique association between lifetime classic psychedelic use and lower sick leave costs in the US would be:

−0.09 × 12 × ($575 billion/264: 230) = −$2.4 billion: $−2.7 billion (2)

Obviously, this is far from an exact number, and builds on major assumptions. However, it gives an approximate estimate about the unique association between lifetime classic psychedelic use and lower sick leave costs: around $2–3 billion a year.

## 4. Discussion

The present study examined the association between lifetime classic psychedelic use and sick leave in the past 30 days in a population-based sample. Findings showed that, in a covariate-adjusted analysis, having used a classic psychedelic, even once, was associated with lower levels of sick leave in the past 30 days. Based on our thought experiment regarding sick leave costs used in this study, lifetime classic psychedelic use was uniquely associated with around $2–3 billion lower sick leave costs. Such findings suggests that classic psychedelics use could potentially lead to reduced sick leave and associated costs in the general population, although no causality can be claimed from this study.

In the first regression model that did not control for any potential sources of confounding, there was a positive association between lifetime classic psychedelic use and sick leave in the past 30 days. In the second regression model, controlling for all confounders used in previous research (i.e., sociodemographic characteristics, risky behavior, and lifetime use of other substances), findings showed that lifetime classic psychedelic use was, as expected, uniquely associated with lower levels of sick leave in the past 30 days. This could potentially be explained by the fact that sociodemographic characteristics have an influence on sick leave independent of ill-health and disease, which is consistent with earlier research [11,12,13,14,15,16]. In further support of this interpretation, the sociodemographic characteristics associated with lower sick leave mirrored those associated with lifetime classic psychedelic users (i.e., being male, white, younger than 65 years of age, having higher education and income) [42]. Regarding confounders of lifetime use of other substances, findings showed that in particular medical drugs such as pain relievers, tranquilizers, and sedatives, but also marijuana, daily cigarettes, and pipe, were associated with higher levels of sick leave, whereas this was not the case for lifetime use of classic psychedelics. A possible explanation for this is that medical drug use among people with pre-existing health problems influenced the association between classic psychedelic use and sick leave. Taken together, these findings are in line with a recent study among Norwegian employees showing that current use of medical drugs and daily smoking were the substance use habits mostly associated with sick leave, whereas current use of illegal drugs and polydrug use (i.e., combination use of several substances) were not associated with sick leave [48].

As the most frequent reason for sick leave is ill-health (e.g., chronic diseases and mental health problems) [13,14,15,16], the findings in the present study may be explained, both directly and indirectly, by better overall health. Indirectly, the acute transcendent experience occasioned by classic psychedelics may induce long-term behavior changes towards a healthy lifestyle [44,45,46] that contribute to better physical as well as mental health. Direct mechanisms that could influence overall health involve improvements on a range of mental health indices such as psychological distress, depression and anxiety [17,29,30,31,32,33,34,35]. Classic psychedelics may also influence factors concerned with positive psychology, such as mindfulness, feelings of purpose and prosociality [50,51], which not only influence mental but also physical health [52,53,54]. In addition, classic psychedelics may have direct immunomodulatory and anti-inflammatory effects of importance for physical health [25,26,27].

## 5. Limitations and Future Research

First, due to the cross-sectional design of this study, inferences regarding causality cannot be made. Second, the study sample did not include institutionalized populations. Third, although controlling for a range of potential confounders, the associations found may be explained by response bias or latent variables that were not controlled for. Fourth, lacking information about use patterns including dose, frequency and context make the findings subject to uncertainties. In order to increase our knowledge of the association between classic psychedelics and sick leave, as well as with other health indicators, it would be of interest for future studies to include measurements on dose and frequency, and also to distinguish between simultaneous polydrug use with regard to different types of classic psychedelics. This can be considered vital as the sensitivity analyses in our study showed that lifetime use of tryptamines and LSD, respectively, but not phenethylamine, was associated with lower sick leave in the past 30 days. Moreover, as the majority of classic psychedelic use within the general population does not take place in research-specific clinical trials, there is a need for studies focusing on the role of context in relation to classic psychedelics. Context, defined as “the interactions between pharmacological, subjective, and social and structural elements” [55], relates to the influence of peoples’ intentions, agency, rational choice and expectations (set) as well as different environmental, situational, social, and structural conditions in which classic psychedelic use takes place (setting) on the experiences, and potentially subsequent outcomes, of classic psychedelic use. 

## 6. Conclusions

Absenteeism from work due to illness, and related costs, have increased steadily during the past decades. In recent years, there has been a reemergence of research on the therapeutic effects of classic psychedelics showing associations with both physical and mental health. However, the association between classic psychedelics and sick leave has so far remained unknown. The findings in the present study revealed that lifetime classic psychedelic use was uniquely associated with lower levels of sick leave in the past 30 days, when controlling for sociodemographic characteristics, risky behavior, and lifetime use of other substances. This demonstrates the need for future studies that investigate potential causal pathways of classic psychedelics on sick leave and evaluate possible mechanisms.

## Figures and Tables

**Table 1:** Unstandardized regression coefficients (B), 95% confidence intervals (CI), and *t*-values (*t*), for lifetime classic psychedelic use (LCPU) on sick leave during the past 30 days (Model 1, *N* = 407,717; Model 2, *N* = 406,881).

		Model 1		Model 2	
		B (CI)	*t*	B (CI)	*t*
Variables					
*LCPU (1 = yes/0 = no)*					
		0.11 (0.07−0.14) **	6.19	−0.09 (−0.15–−0.04) **	−3.45
Control variables:					
*Age (1 = 18–25 Reference Cat)*					
2 26–34				0.02 (−0.01–0.06)	1.20
3 35–49				−0.03 (−0.06–0.01)	−1.52
4 50–64				−0.01 (−0.06–0.04)	−0.33
5 65 or older				−0.06 (−0.15–0.04)	−1.20
*Gender (1 = Male Reference Cat)*					
Female				0.15 (0.11–0.18) **	8.14
*Ethnoracial identity (1 = White Reference Cat)*					
2 (NonHisp Black/AfroAm)				0.28 (0.22–0.33) **	10.35
3 (NonHisp Native Am)				0.36 (0.16–0.55) **	3.62
4 (NonHisp Native/Other Pacific Isl.)				0.53 (0.18–0.88) **	3.06
5 (NonHisp Asian)				0.05 (−0.02–0.12)	1.45
6 (NonHisp Mixed)				0.29 (0.10–0.48) **	3.08
7 (Hispanic)				−0.02 (−0.06–0.02)	−1.14
*Educational level (1 = Less than high school Reference Cat)*					
2 (High school graduate)				−0.03 (−0.09–0.03)	−1.12
3 (Some college)				−0.11 (−0.17–−0.05) **	−3.57
4 (College graduate)				−0.24 (−0.30–−0.17) **	−7.38
*Annual household income (1 = $20,000 or less Reference Cat)*					
2 ($20,000–49,999)				−0.06 (−0.11–−0.01) *	−2.58
3 ($50,000–74,999)				−0.15 (−0.20–−0.09) **	−5.73
4 ($75,000 or more)				−0.20 (−0.25–−0.15) **	−7.93
*Marital status (1 = Married Reference Cat)*					
2 (Widowed)				0.03 (−0.13–0.18)	0.38
3 (Divorced or separated)				0.10 (0.04–0.15) **	3.78
4 (Never been married)				0.03 (−0.01–0.08)	1.60
*Engagement in risky behavior (1 = Never reference Cat)*					
2 (Seldom)				−0.04 (−0.08–0.00) *	−2.27
3 (Sometimes)				0.02 (−0.02–0.07)	0.97
4 (Always)				0.08 (−0.02–0.17)	1.68
*Lifetime use of…(1 = yes/2 = no)*					
Cocaine				0.02 (−0.03–0.07)	0.77
Other stimulants				−0.02 (−0.08–0.03)	−0.86
Sedatives				0.19 (0.12–0.26) **	5.59
Tranquilizers				0.16 (0.11–0.21) **	6.64
Heroin				0.01 (−0.11–0.13)	0.22
Pain relievers				0.21 (0.17–0.24) **	12.43
Marijuana				0.07 (0.03–0.10) **	3.87
Phencyclidine (PCP)				0.19 (0.06–0.32) *	3.00
3,4 methylenedioxy-metamphetamine (MDMA/ecstacy)				0.02 (−0.03–0.08)	0.86
Inhalants				−0.04 (−0.09–0.01)	−1.72
Smokeless tobacco				0.00 (−0.04–0.04)	−0.03
Pipe				0.06 (0.01–0.11) *	2.29
Cigar				−0.04 (−0.07–0.00)	−2.04
Daily cigarettes				0.08 (0.04–0.12) **	4.08
*Age of first alcohol use (1 = 1–12 years Reference Cat)*					
2 (13–19 years)				−0.10 (−0.17–−0.03) *	−2.75
3 (20 or more years)				−0.07 (−0.15–0.01)	−1.69
4 (Never used)				−0.08 (−0.17–0.01)	−1.88
R^2^	0.01			0.01	
*F*	38.31 **			8.72 **	

Lifetime classic psychedelic use (LCPU). * *p* < 0.05; ** *p* < 0.01.

## Data Availability

The NSDUH public use data files are available on their homepage.
